# *NFATc1* is Suppressed in Tumor Microenvironment of Hodgkin Lymphoma

**DOI:** 10.31557/APJCP.2021.22.6.1943

**Published:** 2021-06

**Authors:** Krisna Murti, Neti Neti, Nyiayu Fauziah Kurniawati, Ika Kartika, Riana Sari Puspita Rasyid, Zen Hafy

**Affiliations:** 1 *Department of Anatomic Pathology Faculty of Medicine Universitas Sriwijaya, Indonesia. *; 2 *Department of Histology Faculty of Medicine Universitas Sriwikaya, Indonesia. *

**Keywords:** Hodgkin lymphoma, tumor microenvironment, NFATc1, CD163, PD-L1

## Abstract

**Objective:**

The aims of this research are to evaluate the expression and distribution of *NFATc1* in tumor microenvironment of Hodgkin lymphoma.

**Methods:**

Twenty-eight cases of Hodgkin lymphoma were selected. Clinicopathological data of age, gender, location and subtypes were obtained. Immunohistochemistry was performed to the all cases by using anti-CD163, anti-NFATc1 and anti-PD-L1 antibodies. All protein expression was calculated by using Image J software.

**Results:**

Nuclear expression of *NFATc1* was not observed in Hodgkin cells neither in TAM nor in small lymphocytes surrounding Hodgkin cells in all the samples, this meant that *NFATc1* showed negative nuclear expression in almost all these cells. Cytoplasmic expression of *NFATc1* was observed in small lymphocytes surrounding tumor cells. While there were only few small lymphocytes which were located far from tumor cells showed nuclear expression of *NFATc1*. Meanwhile, 57.14% samples showed high density of TAMs CD163+, and 50% tumor cells as well as 50% TAMs exhibited positive *PD-L1* expression. In addition, all macrophages did not have *NFATc1* expression both in their nuclei and in their cytoplasm.

**Conclusion:**

*NFATc1* was suppressed both in Hodgkin cells and inflammatory cells surrounding the tumor cells. This condition may contribute to progressivity and aggressiveness of the diseases. Therefore, certain mechanisms to reactivate functional *NFATc1* in HL tumor microenvironment may be necessary; hence, the tumor cells are able to be eradicated by patient’s immune mechanisms.

## Introduction

From early development to tumor progression and then metastasis, tumor cells are counter acted by numerous types of tumor microenvironment (TME) elements i.e., stromal factors and immune cells. Therefore, beside the other factors, TME is also an essential component in determinating tumor behavior and prognosis (Kim and Bae, 2016). Numerous markers and therapeutic strategies were developed based on TME context.

In 2018, Hodgkin lymphoma (HL) incidence of new cases were around 79,990 with number of deaths were circa 26,167 (Bray et al., 2018). The incidence of HL varies considerably by age, sex, ethnicity, geographic location and socioeconomic status, and its rates are higher among males and in developed countries, but lower in Asian population. Meanwhile, mortality rates were lower in underdeveloped and higher developing regions (Zhou et al., 2019; Salati e al., 2014). Indonesia ranks 25^th^ in incidence of HL (Ferlay, 2013). Young population at ages 15 to 25 years are mostly affecting by HL with higher incidence (Bigenwald et al., 2017). Despite its relatively low incidence and its low lifetime risk, HL comprises 15% of all cancers in young adults with a high impact on quality of life (Salati et al., 2014). 

HL is a curable disease; more than 90% cure rate for patients with early disease and in more than 70% patients with advanced disease (Shanbhag and Ambinder, 2018). The crucial point is to recognize high-risk patients who will relapse after initial therapy. Therefore, identifying these high risks patients by characterization of pathobiological and clinical prognostic factors then followed by designing properly novel treatment strategies with minimal treatment toxicities is demanding.

Morphologic characteristic of HL is heavily infiltrating inflammatory cells surrounding tumor cells as its tumor microenvironment (Calabretta et al., 2019). In classical HL (cHL) cells NF-кB is constitutively activated (Weniger and Küppers, 2016), however the exact factors regulate its microenvironment is still unclear. Latest findings revealed that abundant component cellular and humoral generated by interaction of Hodgkin cells with their environment, which might contribute to the characteristic background inﬂammatory cells (Calabretta et al., 2019).

Macrophages are the other types of inflammatory cells observed heavily infiltrate the background of Hodgkin cells. Unlike *PD-L1* expressed on tumor cells, *PD-L1* expressed on macrophages is able to protect macrophages from destruction by T cells (Singhal et al., 2019). In addition to this, other studies showed that *PD-L1* in macrophages inducing T cell anergy and M2 polarization (Lu et al., 2019).

Known as an essential transcription factor in many physiologic systems comprising immune cells (Vaeth and Feske, 2018), including in regulation of PD-1 activation (Oestreich et al., 2008), nuclear factor of activated T cell (*NFATc1*) has roles in tumor microenvironment (Li et al., 2018; Gholami et al., 2017). *NFATc1* may contribute to the molecular pathways entailed in tumor microenvironment of HL, which, then both promote to HL progression and worsen prognosis. 

The aims of this research are to evaluate the expression and distribution of *NFATc1* in tumor microenvironment of Hodgkin lymphoma. Together our results may identify *NFATc1* as promising target for alternative novel marker of prognostic and or predictive factors of Hodgkin lymphoma.

## Materials and Methods

*Patient data*

Initially, we collected 44 cases of Hodgkin lymphoma diagnosed based on the 2016 World Health Organization classification (Swerdlow et al., 2017) from January 2014 to November 2019 at Department of Anatomic Pathology, Faculty of Medicine University of Sriwijaya, Dr. Mohammad Hoesin Hospital, Palembang, Indonesia. After careful selection based on quality of fixation and processing which can be assessed by carefully examined the HE and IHC slides, 28 cases were obtained as samples. Clinicopathological parameters i.e., age, gender of patients, subtypes, and location of tumors were attained from patient’s pathology records. Ethical committee approval from Faculty of Medicine University of Sriwijaya was also attained.

*Immunohistochemical analysis*

The paraffin blocks of selected HL cases were retrieved from the archives. Immunohistochemical staining was conducted using manual system according to standard immunohistochemical protocol of our lab. The analyses were validated using appropriate negative and positive controls by using several tissue blocks consisting of tonsil, appendix, melanoma and breast cancer tissues. After sectioning, the blocks were dried in a lab heating and drying followed by deparaffinization and rehydration. Then antigen retrieval was performed by treating the slides in a microwave in citrate buffer. After blocking step the tissues were incubated for 60 minutes with primary antibody *NFATc1* (clone 7A6, dilution 1:200, BD Pharmingen, Franklin Lakes, New Jersey), CD163 (clone 10D6, rabbit, monoclonal, dilution 1:100, thermo fisher, USA) and *PD-L1* (clone SP142, dilution 1:100, Abcam, Cambridge, MA). Lastly, the slides were covered with mounting medium and coverslips. Stained tissues and all pictures were analyzed and captured using Olympus BX41 (Tokyo, Japan) couple with camera (12MP1/1.7” Sony Exmor CMOS Sensor, Beta Industrial Digital Camera, China) at a ×400 magnification. 

*Expression of NFATc1, CD163, and PD-L1*

The positive expression of all antibodies was determined disregard staining intensity, since the later was most likely influenced by inconsistency of tissue fixation and processing. *NFATc1* positive expression was determined in nuclei of tumor cells as well as in lymphocytes and macrophages surrounding tumors. Positive expression of CD163 was calculated in membrane and or cytoplasm of macrophages around tumor cells. In addition, positive expression of *PD-L1* was counted in membrane of Hodgkin tumor cells and macrophages around tumor cells. Image J was used to quantify the numbers of protein expression of *NFATc1*, *CD163*, and *PD-L1*.

*Density of NFATc1, CD163, and PD-L1*

Reactivity of every antibody was differentiated into high and low density based on cut-off point obtained from median value. At the beginning the most concentrated five locations containing brown staining either *NFATc1*, or* CD163* or *PD-L1* were selected under low power field (100x). Then among these areas, the five most densest focuses were carefully chosen and photographed under high magnification (400x). By using image J software, the all cells expressed either *NAFTc1*, or *CD163*, or* PD-L1 *were calculated and noted. Of these five areas, the average was counting by using excel. The median of all samples of each antibody was considered as a cut-off point for differentiation of *NAFTc1*, or* CD163*, or *PD-L1* expression into high or low density. 

*Statistical Analysis*

Since *NFATc1* expression was negative in the evaluated area of all the samples, the statistical analysis was not performed. 

## Results

*Patients Characteristics*

Among 28 total samples, our data only have one case of NLPHL and 27 cHLs. The age was differentiated into five groups i.e., under 20 years (10.7%), between 20 to 29 years (25%), between 30 to 39 years (10.7%), between 40 to 49 years (28.6%) and after 50 years (25%). More patients in the ages of 40 to 49 years suffer from HL. Males suffer from HL more than that in females (57.1%). Tumor masses were mostly found in head and neck (78.6%). Lymphocyte-rich cHL was the subtype which mostly observed (57.1%) among others (Table 1).

*Immunohistochemistry*

*NFATc1, CD163 and PD-L1*

**Figure 1:**
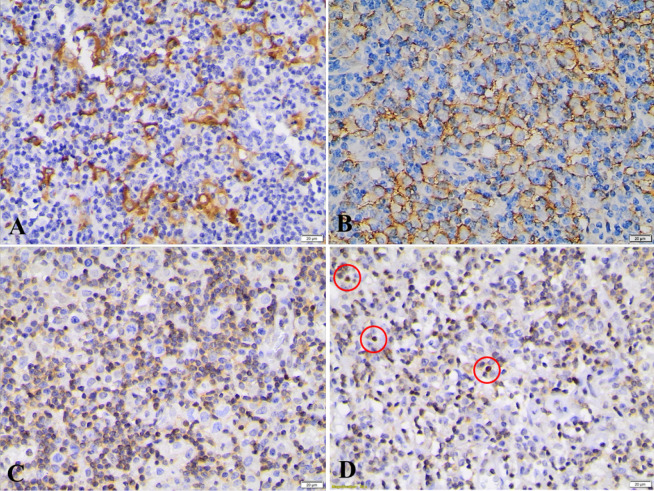
The Immunoreactivity of CD163, PD-L1 and NFATc1 Proteins of Patient #1. A. Showed various positive cytoplasmic expression of TAMs CD163+. B. Immunoreactivity of PD-L1 in membrane of Hodgkin cells and TAMs. C. Demonstrated various negative nuclear expression of NFATc1 protein in all cells; the only finding was cytoplasmic expression of NFATc1, particularly in small lymphocytes surrounding the Hodgkin cells. D. It can be seen few small lymphocytes showed nuclear expression but far from Hodgkin tumor cells (red circles). Original magnifications ×400

**Figure 2:**
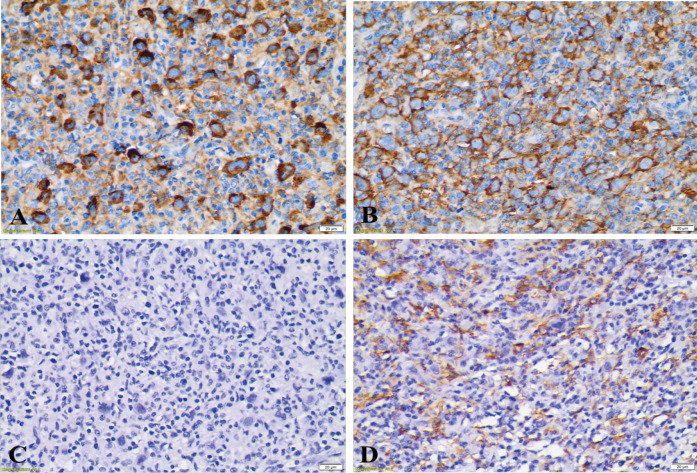
The Immunoreactivity of CD163, PD-L1 and NFATc1 Proteins of Patient #2. A. Varied immunoreactivity of PD-L1 in membrane of Hodgkin cells and TAMs. B. Positive cytoplasmic expression of TAMs CD163+. C. Negative nuclear expression of NFATc1 protein in all cells. D. Membrane expression of CD163. Original magnifications ×400

Nuclear expression of *NFATc1* was not observed in Hodgkin cells neither in TAM nor in small lymphocytes surrounding Hodgkin cells in all samples (Table 2), this meant that *NFATc1* showed negative expression in almost all these cells. There were only few small lymphocytes showed nuclear expression of *NFATc1* in some patients (Figure 1). These cells were located far from tumor cells, while small lymphocytes surrounding tumor cells have only cytoplasmic expression of *NFATc1* (Figure 1 and Figure 2). Approximately 57.14% samples showed high density of TAMs CD163+. In addition, all macrophages did not have *NFATc1* expression both in their nuclei and in their cytoplasm’s. The expression of *PD-L1* was observed in tumor cells and in TAMs surrounding tumor cells, with similar percentage (50%) both in high and low density in those two types of cells (Table 2).

**Table 1:** Patient Characteristics

Clinical features	N (28)	%
Age (years)		
<20 years	3	10.7
20-29	7	25.0
30-39	3	10.7
40-49	8	28.6
≥50 years	7	25.0
Gender		
Male	16	57.1
Female	12	42.9
Location		
Head-neck	22	78.6
Body	2	7.1
Extremities	4	14.3
Subtypes and Variant		
NLPHL	1	3.6
CHL		
NSCHL	4	14.3
LRCHL	16	57.1
MCCHL	7	25.0
LDCHL	0	0.0

**Table 2:** The Expression of NFATc1, CD163 and PD-L1

Antibodies	Lymphocytes		MΦ		Tumor cells	
	H	L	H	L	H	L
NFATc1	0	0 (0%)	0	0	0	0
	0%		0%	0%	0%	0%
CD163	-	-	16 (57.14%)	12 (42.86%)	-	-
PD-L1	-	-	14 (50%)	14 (50%)	14 (50%)	14 (50%)

N, 28; MΦ, macrophages; H, high; L, low

**Figure 3:**
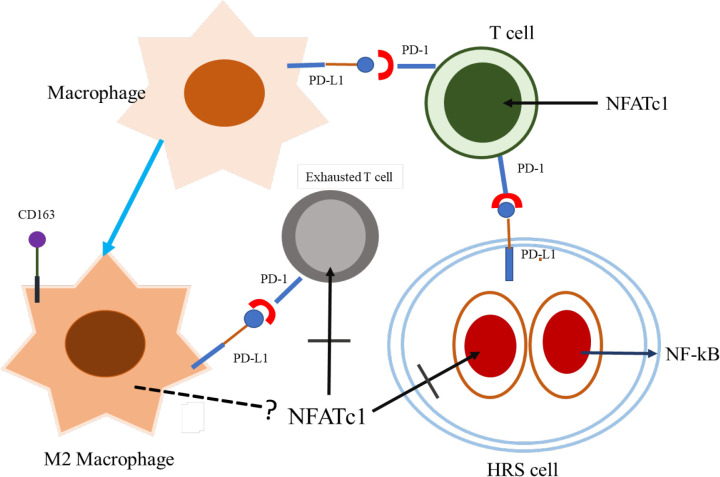
NFATc1 Loss in Hodgkin Cells and CTLs may Contribute to the Molecular Pathways Entailed in Tumor Microenvironment of HL. Macrophages and T cells are heavily infiltrate the background of HL. PD-L1 in macrophages inducing T cell anergy and M2 polarization. While NFATc1 is not expressed in Hodgkin cells caused by epigenetic silenced mechanism, NF-кB is constitutively activated in these cells. The exact regulation of this microenvironment is still unclear and need to be elucidated

## Discussion

Recent studies have identified the impact of non-neoplastic cells on disease pathobiology, particularly immunohistochemical studies of cells in the tumor microenvironment. As a result, some biomarkers have identified and translated into clinical practice. The transcription factors NF-κB and NFAT are known as essential factors in activation of B cell lymphocytes (Muhammad K et all., 2014). However, in Hodgkin cells *NFATc1* is not expressed caused by epigenetic silenced mechanism (Akimzhanov et al., 2008), while NF-κB is constitutively active in these tumor cells (Weniger and Kuffer, 2016). Our finding confirmed the results of previous studies (Akimzhanov et al., 2008; Marafioti et al., 2005) that *NFATc1* was not expressed in Hodgkin cells. However, *NFATc1* expression in tumor microenvironment was not discussed in earlier experiments. Our data showed that there were only few small lymphocytes expressed nuclear *NFATc1*, but these cells were situated far from tumor cells. While small lymphocytes which located closed to Hodgkin cells only showed cytoplasmic *NFATc1* expression, none of them have *NFATc1* nuclear expression. 

It is known that T cell lymphocytes surrounding Hodgkin cells exhibited unusual phenotypic and functional characteristics may be due to impairment of their regulation (Fozza and Longinotti, 2011). Initially, the lymphocytes were most likely activated and induced to come to tumor microenvironment, as can be seen from Figure 1 that few lymphocytes located far from tumor cells which showed nuclear expression of *NFATc1* suggesting that *NFATc1* is essential for T and B lymphocytes activation, homeostasis and differentiation (Vaeth and Feske, 2018). Most Hodgkin tumor cells were surrounded by T-lymphocytes expressing PD-1 (Ilcus et al., 2017). The expression of PD-1 receptor driving in decreased activation of *NFATc1* (Sharpe and Pauken, 2018), thereby, this mechanism is one factor that was most likely led to down regulation of *NFATc1 *in lymphocytes surrounding tumor cells in our samples, yet the exact mechanism is still unclear. This mechanism benefits for survival of tumor cells since TILs expressing PD-1 impaired their effector functions by displaying exhausted phenotype (Thommen and Schumacher, 2018; Ilcus et al., 2017). Future study is needed to unravel how the precise mechanisms control the silencing of *NFATc1* in tumor microenvironment of HL. 

Increased TAMs CD163+ was correlated to unfavorable outcomes (Guo et al., 2016). We did not have any data of patient survival; therefore, we were unable to correlate the presence of TAMs to our patient outcomes. However, here we would like to know whether *NFATc1* may have roles in activation of TAMs CD163+ in tumor microenvironment of HL. In fact, both the nuclear and cytoplasmic *NFATc1* expression in TAMs CD163+ were not observed. Down regulation of *NFATc1* in TAMs and Hodgkin cells may result in T cells anergy, thus, promotes tumor progression. The exact role of *NFATc1* in recruitment and or activation of TAM in tumor milieu is unclear 

In our samples, half patients showed high density of *PD-L1* in tumor cells and the same percentage as in macrophages around tumor cells. Patients with high density of tumor cells expressing *PD-L1*, also showed high density of TAMs CD163+ with *PD-L1* expression. This suggests TAMs have important roles in microenvironment of Hodgkin lymphoma. However, we have no information about survival data, hence, we cannot correlate the expression of *PD-L1* in those cells with patient survival, thus, patient prognosis. The expression of *PD-L1* in Hodgkin cells usually correlated to worse prognosis (Jalali et al., 2019). While the expression of *PD-L1* in macrophages could lead to T cell anergy and M2 polarization, indicating that high levels of *PD-L1* expression in macrophages were in accordance with an immunosuppressive tumor environment and decreased anti-tumor immunity (Lu et al., 2019: Jalali et al., 2019; Gordon et al., 2017). Together the expression of *PD-L1* in Hodgkin tumor cells and TAMs lead to worse prognosis of Hodgkin lymphoma patients (Karihtala et al., 2020). It was possible that silencing of *NFATc1* expression may contribute to HRS cells to become immortal and correlated to inferior outcomes. This hypothesis should be investigated by further experiments. Understanding the exact mechanism of *NFATc1* regulation in TME could lead to development of therapeutic pathway by restoring antitumor immunity.

In conclusion *NFATc1* was suppressed both in Hodgkin tumor cells and inflammatory cells surrounding the tumor cells. This condition may contribute to progressivity and aggressiveness of the diseases (Figure 3). Therefore, certain mechanisms to reactivate functional *NFATc1* in cHL tumor microenvironment may be necessary; hence, the tumor cells are able to be eradicated by patient’s immune mechanisms. 

## Author Contribution Statement

All authors read, critically reviewed and approved the final manuscript. KM designed the study, analyzed all data, drafted the manuscript, conducted pathologic interpretation and lymphoma diagnosis also edited the ﬁnal manuscript text. NN and NFK assisted the experimental process. IK contributed to lymphoma diagnosis. RPR and ZH contributed to the preparation of the manuscript, editing and review. This manuscript is a part of an approved student thesis
